# Acceptability and Implementation of a Primary Care Health Check for Autistic People: Findings From Evaluation Questionnaires and Interviews

**DOI:** 10.1177/13623613261433106

**Published:** 2026-06-16

**Authors:** Hannah Merrick, Helen Taylor, Barry Ingham, Tracy Finch, Sarah Al-Ashmori, Ruby Herrington, Clare Scarlett, Carole Buckley, Sally-Ann Cooper, Cristina Fernandez-Garcia, Shona Haining, Rhianna Lees, Nicholas Lennox, Sebastian Moss, Tim Nicholls, Christina Nicolaidis, Malcolm Osborne, Dora M Raymaker, Tomos Robinson, Anna Urbanowicz, James MS Wason, Colin Wilson, Jeremy R Parr

**Affiliations:** 1 Newcastle University, UK; 2 Cumbria, Northumberland, Tyne and Wear NHS Foundation Trust, UK; 3 Northumbria University, UK; 4 NHS North East and North Cumbria Integrated Care Board, UK; 5 Royal College of General Practitioners, UK; 6 University of Glasgow, UK; 7 The University of Queensland, Australia; 8 National Autistic Society, UK; 9 Portland State University, USA; 10 Oregon Health & Science University, USA; 11 The Kayaks Support Group, UK; 12 Deakin University, Australia

**Keywords:** acceptability, autism, health checks, implementation, primary care

## Abstract

Autistic people experience higher rates of physical and mental health conditions and face barriers to accessing healthcare. This study evaluated the acceptability and implementation of a co-designed primary care health check for autistic people (18 years+), delivered within a randomised controlled trial in Northern England investigating clinical and cost-effectiveness. Following health checks, all autistic people, carers/supporters, and clinicians were invited to complete evaluation questionnaires and a subset invited for an interview. Eighty-one autistic people, 11 carers/supporters, and 18 clinicians returned questionnaires and were descriptively analysed; 44 participants were interviewed. Findings demonstrated that the pre-appointment questionnaire and health check appointment were reported to be acceptable, valued and beneficial by all groups. Carers/supporters reported positive impacts of their role in reducing anxiety and supporting communication. Interview analysis, informed by Normalisation Process Theory, highlighted important factors to consider for implementation of the health check at scale. This included the importance of providing clear guidance and resources ahead of the health check and ensuring enough funding and capacity in primary care. Health checks can be delivered to autistic people in local neighbourhoods to address heath inequalities. Health checks require adequate resources, staff training, and integration into care systems to ensure successful implementation and sustainability.

**Lay Abstract:**

**Improving healthcare for autistic people: a study of a new primary care health check**

Autistic people often face more health problems and shorter average life expectancy compared to non-autistic people. They can also find it harder to access healthcare that meets their needs. To help with this, researchers developed a new health check specifically for autistic people to be used in general practice settings. This health check included a pre-appointment questionnaire to help prepare for the visit and a longer appointment with a trained clinician. This study looked at how acceptable the health check was for autistic people, their carers/supporters, and clinicians, and what factors will help it work well in practice. It used questionnaires and interviews to collect views from autistic people who received the health check, clinicians who delivered it, and carers/supporters who attended the appointment. Most autistic people thought the health check was helpful and they would attend one again. The pre-appointment questionnaire helped people share important information, although some found it difficult to complete and some people needed support to complete it. Carers/supporters also found the process helpful in supporting the autistic adult and improving communication. Clinicians said they were able to put adjustments in place, use the clinician health check template, and found the extra time and structure the health check provided useful. However, they mentioned needing more resources and support to make it work in everyday practice. The interviews showed that autistic people, carers/supporters, and clinicians thought the health check is a good idea and should be offered more widely. It is important to make sure autistic people understand what to expect from the health check and that they can ask for adjustments to meet their needs. For the health check to work well long term, general practice settings will need funding, staff training in autism awareness, and clear systems for delivering the health checks.

## Introduction

Autistic people experience higher rates of physical and mental health conditions compared to non-autistic people, independent of the presence of an intellectual disability (Hand et al., 2020; Rydzewska et al., 2021; Ward et al., 2023). These disparities contribute to increased morbidity and reduced average life expectancy in autistic people (Hirvikoski et al., 2016; Kennedy et al., 2022; Schendel et al., 2016). Despite greater overall healthcare utilisation, autistic people often face unmet healthcare needs and reduced access to preventive health services (Gilmore et al., 2022; Zerbo et al., 2019). Barriers to effective healthcare access include sensory sensitivities in clinical environments (Mason et al., 2019; Smith-Young et al., 2025), challenges with patient-provider communication (Nicolaidis et al., 2015), limited autism-specific training among clinicians (Unigwe et al., 2017), and cognitive difficulties such as executive functioning challenges that affect appointment attendance and follow-up care (Bradshaw et al., 2019; Doherty, 2020; Nicolaidis et al., 2016). In the United Kingdom (UK), reasonable adjustments (changes to services, procedures or environments) to healthcare services are sometimes available; however, evidence suggests a gap between requested adjustments and those received (Brice et al., 2021).

A systematic review of interventions to improve healthcare experiences and access for autistic people found most interventions place the burden on autistic people themselves; few focused on changing provider practices (Walsh et al., 2023). Tailored assessment approaches to support autistic people have been developed. In Australia, the Comprehensive Health Assessment Program (CHAP; Lennox et al., 2016), originally designed for individuals with intellectual disabilities, was adapted for autistic people with co-occurring intellectual disabilities. In the United States, the Academic Autism Spectrum Partnership in Research and Education (AASPIRE) created a Healthcare Toolkit including the online Autism Healthcare Accommodations Tool (AHAT), to help autistic people generate personalised accommodation reports for their providers (Nicolaidis et al., 2020). Preliminary findings showed the AASPIRE Healthcare Toolkit reduced barriers to care, improved patient–provider communication, and increased healthcare self-efficacy (Kang et al., 2022). At a 2017 research priority-setting workshop held at Newcastle University, UK, autistic people, supporters, and clinical staff prioritised research to create and evaluate a health check for autistic people (Warner et al., 2019).

Annual health checks for individuals with intellectual disabilities have been found to improve health outcomes by identifying unmet needs and prompting appropriate interventions (Cooper et al., 2014; Kennedy et al., 2022; Lennox et al., 2010; Robertson et al., 2014). These are available to autistic people with an intellectual disability, but not those without. Whether health checks should be offered to all autistic people has been a focus of recent UK and international healthcare policies. The UK NHS Long Term Plan and the National Strategy for Autistic Children, Young People, and Adults advocated for the development and evaluation of health checks specifically designed for all autistic people (Department of Health and Social Care & Department for Education, 2021; National Health Service, 2019). Despite policy recommendations, implementing annual health checks for autistic people faces challenges. A recent study in England by Davies et al. (2024) found that while clinicians recognise the potential benefits of health checks for autistic people, practical challenges such as limited resources, time constraints, and a lack of autism-specific training hinder effective implementation.

This study was completed as part of a programme of research to design and evaluate a health check for autistic people (see supplementary file 1). The first stage aimed to understand the barriers and facilitators of healthcare access for autistic people and gathered ideas for the development of a health check for autistic people (Mason et al., 2022). The second stage co-designed an autism-specific health check, including a training package for primary care staff (about autism and conducting the health check); a pre-appointment questionnaire (PAQ) for completion before the health check that informed reasonable adjustments and health and social concerns; and a clinician template that addressed physical health, mental well-being and other support needs (Taylor et al., 2023). The third stage was a randomised controlled trial (RCT) of the health check’s clinical and cost-effectiveness (Parr et al., 2024). Alongside this, a mixed-methods evaluation of the health check acceptability and key features needed for effective implementation was carried out, which is the focus of the analysis reported below. In implementation science, acceptability refers to how stakeholders perceive an intervention in terms of its suitability, usefulness, and overall satisfaction (Proctor et al., 2011). Understanding acceptability and implementation from the perspectives of both autistic people and primary care staff is critical to ensure that the health check is not only clinically effective but also feasible, meaningful, and sustainable in practice. Acceptability influences engagement, informs necessary adaptations, and helps identify barriers and facilitators to uptake, which is particularly important given the diversity of needs within the autistic community and the operational pressures faced by primary care services.

## Methods

### Ethical Approval

This study received ethical approval from the North Wales Research Ethics Committee (Wales REC 5) and the NHS Health Research Authority (ref: 21/WA/0196; IRAS Project ID: 272808). Informed consent was obtained from all study participants. The trial was registered with ISRCTN 30156776 (https://www.isrctn.com/ISRCTN30156776) and the NIHR Portfolio.

### Participants and Recruitment

One hundred and ten autistic people, with and without intellectual disabilities, were recruited to the health check arm of the RCT from 26 primary care practices across three areas in Northern England who were not currently delivering or trialing a health check for autistic adults (see supplementary file 2 for flow of participants through the trial). Ninety-five received a health check. All autistic people recruited had a clinically confirmed autism diagnosis; were aged 18 years or over; had not received an NHS Learning Disability annual health check in the last 12 months or were not anticipated to attend one in the next 3 months; and were not in the final stages of a terminal illness. People who could consent to participate were included, and people who were unable to consent for themselves participated if they had a relative or supporter who could consent for them, in keeping with England’s Mental Capacity Act. Autistic adults received an information pack from their primary care practice which included an information sheet (as well as short and easy-read versions) and an expression of interest form to return to the research team. In consenting to the RCT, autistic people agreed to complete questionnaires, be invited to a health check, be invited to complete an evaluation form after the health check, and be invited to take part in an interview. As part of the baseline measures collected for the RCT, autistic adults completed a bespoke demographic form (which included questions about personal characteristics, their living situation, employment and education, learning ability, support needs and health conditions) and the Social Responsiveness Scale (SRS; Constantino & Gruber, 2012).

All clinicians (a healthcare staff member providing care, e.g. GP, nurse) who delivered the health check and non-clinical practice staff (e.g. administrators) supporting delivery were invited to participate and received an information sheet, consent form, and demographic questionnaire. Non-clinical staff were identified by the research team and clinicians. Clinicians consented to complete evaluation questionnaires and take part in an interview. Non-clinical practice staff consented to be interviewed only. At site setup, demographic details were also collected about the primary care practices, including the size of practice (> or ≤10,000 patients), geographical areas covered by the practice, and if the practice was based in an urban, rural or mixed area.

All carers/supporters who attended a health check appointment with an autistic adult were invited to participate. The clinician delivering the health check provided an information pack (information sheet, contact form, consent form and demographic questionnaire) with a pre-paid envelope to return the documents to the research team. Consent covered completing questionnaires and agreeing to be invited to take part in an interview.

## Procedures

### Health Check Evaluation Questionnaire

Three bespoke Health Check Evaluation Questionnaires (HCEQs) were developed in collaboration with the consortium group, which consisted of international researchers, autistic people, carers/supporters, and primary care clinicians. The version for autistic people focused on the PAQ and health check appointment; for carers/supporters the focus also included how they supported the autistic adult and what they gained from the health check. Clinicians reported on delivering the health check, using the health check clinician template (which guided topics to cover) and training received. The questionnaires included closed-ended and open-text questions.

All autistic people, carers/supporters who attended a health check appointment, and the clinicians who delivered the health check, received the HCEQ and a pre-paid return postal envelope. The HCEQ invitation letter contained instructions about how to request a link to complete it online if preferred.

### Interviews

Semi-structured interviews explored views on the health check, including aspects relating to the implementation of the health check, such as availability of reasonable adjustments and barriers or facilitators to implementation in the NHS, as well as the acceptability of the health check (see supplementary file 3 for Topic Guides).

Autistic people who received a health check were purposively sampled based on primary care practice, age, gender, and responses from their HCEQ (i.e. positive and negative responses). Gender and age were selected to reflect potential variations in healthcare access, communication preferences, and support needs among autistic people. Including participants from different primary care practices allowed exploration of how the delivery model used, practice size, and geographical context (urban, rural, mixed) may influence the acceptability and implementation of the health check. Selected participants were invited to participate via their preferred contact method and received another copy of the trial information sheet as a reminder of interview activities and data handling.

All carers/supporters who returned consent forms, all clinicians involved in delivering the health check and all non-clinical practice staff who supported health check delivery were invited to take part in an interview.

Interviews with each group were carried out in parallel within 2 months of the health check being received or delivered. Interviews took place at a time convenient for the participant, 1:1 either via video or telephone call with a researcher (H.M., H.T.), following the participant preference. One autistic adult and one non-clinical practice staff requested to complete the interview via email exchange. Participants were given the option to see the questions in advance. All participants confirmed consent at the beginning of the interview. Interviews were recorded, transcribed, and anonymised for qualitative analysis. Autistic adults received vouchers for their overall participation in the trial as a token of thanks. Clinical and non-clinical staff had their time taking part in interviews reimbursed through research costs.

#### Data Analysis

HCEQ data were analysed separately for each group. Categorical variables were summarised by frequency and percentage. Continuous data was summarised by the mean and standard deviation and/or median, interquartile range (IQR) and range, as appropriate. Open-text responses were categorised and summarised. Within-arm comparative analysis was conducted on selected items of the HCEQ autistic peoples’ version, comparing responses by gender, age, SRS-2 (Constantino & Gruber, 2012) total score, and the number of health conditions identified at baseline. Within-group comparative analysis was also conducted on certain items of the HCEQ clinician’s version comparing responses of clinicians from small (<10,000 patients) versus large (⩾10,000 patients) practices and from different environments (urban vs rural vs mixed). Responses were summarised descriptively by group and compared using Fisher’s exact test.

Interview transcripts were imported into NVivo 14 for analysis. Initial analysis used Braun and Clarke’s Reflexive Thematic Analysis (Braun & Clarke, 2019). Three researchers (H.M., H.T., and R.H.) followed the process of familiarisation with the data, initial coding, theme development, refinement, and creating theme narratives. The analysis was iterative, with ideas and themes evolving through engagement with the data and through discussions and reflections by the researchers carrying out the analysis and the wider research team. The inductively developed codes and themes were grounded in the data, and a realist epistemological stance was adopted.

Initial inductive coding revealed that data had been generated on the range of barriers and facilitators to the health check delivery and data around the implementation of health checks for autistic people. As a higher-level analysis, initial inductively developed coding was then deductively mapped onto Normalisation Process Theory (NPT) constructs (coherence, cognitive participation, collective action, and reflexive monitoring (Murray et al., 2010)) to develop the understanding of the work done by each stakeholder group (autistic people, carers/supporters, and practice staff) to implement the health checks into primary care. Throughout analysis, there were cycles of mapping and checking the alignment of codes to the four NPT constructs with the wider research team to check and discuss analysis and interpretation. Researchers blind to participant group did not receive transcripts to maintain blinding for RCT primary outcome rating.

## Results

### HCEQs

Table 1 presents the demographic information of those who returned an HCEQ. In all, 81/95 (85%) autistic people completed an HCEQ. Eleven carers/supporters (of a known 25 that were reported to attend the health check appointment) returned a completed HCEQ, and 18/23 (78%) clinicians completed at least one HCEQ (individual clinicians completed between 1 and 9 HCEQs; one was completed for each health check they delivered). In total, 71 clinician HCEQs were returned from the 95 health checks completed (75%).

**Table 1.** Demographic Information of Participants Returning an HCEQ.

	Autistic people^a^	Carers/supporters	Clinicians
	N = 81	N = 11	N = 18
Age (years)			
Mean (SD)	34.1 (13.6)	54.3 (8.1)	42.0 (12.5)
Median (IQR)	29.0 (24.0, 42.0)	53.0 (52.0, 58.0)	40.50 (31.0, 56.0)
Range	19.0, 76.0	37.0, 68.0	23.00, 63.00
Gender			
Female	36 (45.0%)	10 (90.9%)	13 (72.2%)
Male	34 (42.5%)	1 (9.1%)	5 (27.8%)
Transgender or non-binary	8 (9.9%)	–	–
Rather not say	2 (2.5%)	–	–
Ethnicity			
White	74 (92.5%)	11 (100.0%)	16 (88.9%)
Asian/Asian British	1 (1.2%)	–	2 (11.1%)
Mixed/multiple	3 (3.8%)	–	–
I’d rather not say	2 (2.5%)	–	–
Current employment status			
Employed full-time	32 (40.0%)	3 (27.3%)	–
Unable to work	18 (22.5%)	1 (9.1%)	–
In education or training	14 (17.5%)	1 (9.1%)	–
Employed part-time	13 (16.2%)	3 (27.3%)	–
Looking for work	4 (5.0%)	–	–
Retired	3 (3.8%)	1 (9.1%)	–
A volunteer	2 (2.5%)	–	–
A carer/have caring responsibilities	–	5 (45.5%)	–
Homemaker	–	–	–
Unknown	–	–	–
Clinical role			
GP	–	–	9 (50.0%)
Research nurse	–	–	4 (22.2%)
Nurse practitioner	–	–	3 (16.7%)
Receptionist/administrator	–	–	1 (5.6%)
Clinical pharmacist	–	–	1 (5.6%)

a Details on one participant are missing as we were not able to identify who completed one HCEQ received.

#### Quantitative Findings

##### Autistic People

Autistic people’s responses are summarised; full details are available in Supplemental Tables S1 and S2. Most autistic people (99%) reported completing the PAQ (56% paper version; 41% online). The majority found the PAQ “okay to complete” (90%); though 41% still reported some “difficulties” with it. The difficulties reported in open text responses included challenges with the topics asked about, the length of the questionnaire, and the understanding of the questions. The majority found the PAQ useful (75%), especially for sharing information prior to the appointment about their communication (58%), current health concerns (56%) and general health and well-being (59%). Self-reported PAQ completion time varied, with 41% reporting 15–30 minutes, and 19% reporting >60 minutes. Two-thirds (68%) thought the completion time was appropriate; 24% found it “a little too long,” and 9% reported it was “much too long.” In open responses, one participant suggested providing example answers for open text questions, and another suggested having versions for people with or without intellectual disabilities.

Regarding the health check appointment, 46% requested reasonable adjustments; of these just under half (46%) reported they were contacted about them beforehand, and 59% reported most or all adjustments were implemented. All those who requested adjustments found them helpful. Nearly one-third of responders (31%) brought someone for support, and all found this beneficial for comfort, communication, and simplifying information. Almost 91% of autistic people said they would attend another health check, and 85% suggested they be annual. Most (69%) said they learned something from the health check, reporting outcomes such as “addressing health concerns,” “starting new medication,” and “being signposted to services like mental health practitioners.”

In a final open-ended question, autistic people described satisfaction with the health check appointment (n = 13), praising the clinician’s manner, the extra time for thorough discussions, and valuing the reasonable adjustments. However, some reported unmet adjustments (n = 2), the appointment not being long enough (n = 2), wanting more preparatory information (n = 1), and more physical checks (n = 1).

A Fisher’s exact test explored associations between age, gender, social communication difficulties (SRS-2), and number of health conditions, with evaluation outcomes (see Supplemental Table S3). No significant differences emerged for items on PAQ usefulness, willingness to attend future health checks, or whether participants learned something from the health check. However, preferences for health check frequency differed by health condition count (p = 0.037), with those reporting more conditions favouring annual checks.

##### Carers/Supporters

Carers/supporters responses are summarised; full details are available in Supplemental Tables S4 and S5. Nearly two-thirds reported helping the autistic person to complete the PAQ (27%) or completing it on their behalf (36%). All but one found it “okay” to complete, and all who assisted believed it was useful for “sharing key information.” Over half (57%) reported that the PAQ caused “difficulties for the autistic adult,” with many providing support to mitigate this. Most (71%) thought the time taken to complete the PAQ was “about right.”

More than half the carers/supporters (55%) helped arrange the health check. In open responses, they reported that their presence was helpful in terms of easing anxiety (64%) and aiding communication/interpreting information (55%). All carers/supporters indicated willingness to attend future health checks, endorsing annual frequency. Most reported “gaining something” from the health check process (82%); however, only 18% thought it improved their own personal quality of life.

Open responses included suggestions to improve the clarity of PAQ questions and knowing the clinician’s identity beforehand. Several carers/supporters praised clinicians’ communication and understanding; one noted the clinician seemed preoccupied with their screen. A final comment praised the thoroughness and the autistic adult feeling heard.

##### Clinicians

Responses from the clinicians are summarised; full details are available in Supplemental Tables S6 and S7. For 39% of health checks, clinicians reported receiving automatic summaries from online PAQs. For the 49% manually preparing summaries when a paper PAQ was returned, this took an average of 23.1 minutes, plus 10 minutes for review. Most clinicians (85%) thought they had “enough time” to review and understand the summaries. Adjustments were requested in 48% of health checks and 82% of clinicians said they were able to implement “most or all” of these. Barriers to implementation included late/same day PAQ submissions, and practical limits such as building constraints or staff availability. The average reported time taken to talk to patients to agree on adjustments and put them in place was 7 and 4 minutes, respectively, with nearly all not requiring any additional staff resource. Health checks averaged 58 minutes (SD = 22.1), with 72% of clinicians reporting that they completed “most sections” of the health check clinician template. Reasons given for uncompleted sections included “prior recent care,” “opting to focus on key concerns,” or “managing appointment length.” In 85% of cases, a health action plan was created, with 77% provided to the autistic adult during the appointment and 8% emailed/posted later. Plans were not created when no new health or social needs emerged, or existing plans covered them.

Fisher’s exact tests compared clinician responses by practice size and environment. Significant differences emerged for health action plan creation, where larger practices were more likely to create plans than smaller ones (p = 0.001), and urban practices were more likely to create plans than rural or mixed settings (p = 0.012) (see Supplemental Table S8).

### Qualitative Findings

#### Participants

Forty-nine autistic people were invited to take part in an interview; 24 (49%) were interviewed (see Table 2). Five of the 11 carers/supporters were interviewed (1 declined, 5 did not respond). Four interviewees were mothers, and one was the wife of an autistic person. Of the 21 clinicians invited for an interview, 11 (58%) agreed; remaining clinicians did not respond (n = 7), declined (n = 1), or were not able to participate for other reasons (n = 2). Five General Practitioners (medical doctors), four practice nurses, one pharmacist, and one research nurse were interviewed. Ten non-clinical practice staff were identified and invited to take part in an interview. Four agreed to be interviewed; two research nurses and two receptionists/administrators.

**Table 2.** Demographic Information of Autistic People Approached about Interview and Participants Interviewed.

	Autistic people approached (n = 49)	Autistic people interviewed (n = 24)	Carers/supporters (n = 5)	Clinicians (n = 11)^a^	Non-clinical staff (n = 4)^a^
Gender					
Female	23 (47%)	12 (50.0%)	5 (100%)	5	3
Male	20 (40.8%)	9 (37.5%)	–	4	–
Transgender, agender or non-binary	5 (10.2%)	2 (8.3%)	–	–	–
Prefer not to say	1 (2%)	1 (4.2%)	–	–	–
Age group					
Under 20	6 (12.2%)	1 (4.2%)	–	–	–
20–29	18 (36.7%)	9 (37.5%)	–	2	1
30–39	8 (16.3%)	4 (16.7%)	–	3	–
40–49	6 (12.2%)	3 (12.5%)	–	4	–
50–59	7 (14.3%)	5 (20.8%)	4 (80%)	–	1
Over 60	4 (8.3%)	2 (8.4%)	1 (20%)	–	1
No. of sites recruited from	19/26	11/26	5/26	11/26	4/26
PAQ completion preference					
Online	32 (65.3%)	17 (70.8%)	–	–	–
Paper	17 (34.7%)	7 (29.2%)	–	–	–

a Demographic information not returned by two clinicians and one non-clinical staff.

#### NPT Analysis

Autistic people, carers/supporters, and primary care staff spoke about the positive impact of the health check on the health and well-being of autistic people and their engagement with primary healthcare services. Each group also discussed the challenges for the implementation of health checks for autistic people in primary care. The findings described below highlight some of the considerations required for health service implementation of health checks for autistic people based on the four constructs of NPT (see Table 3 for further detail and supporting quotations).

**Table 3.** Mapping of Interview Data onto the Normalisation Process Theory Constructs.

NPT construct	Key findings	Supporting quotations
**Coherence** *Making sense of what the intervention is and its relevance*		
	• All stakeholders recognised the aim is to prevent autistic people “slipping through the net” and improve access to primary care. • Opportunity to see clinician and discuss health and well-being needs. • Helpful for those who do not regularly seek out primary care support. • Comprehensive review of physical and mental health. • Some autistic people not clear on scope of health check. • Clinicians raised which groups of autistic people should receive a health check .• Autistic people viewed it as different to usual care/access to primary care. • Autistic people and clinicians described more time to discuss needs and space to be listened to.	“Having that regular contact even annually is something that’s making sure that somebody’s mental health isn’t getting worse.” (PU_CS_001) “They are a population of people that need better access. That’s what, and we’re trying to be proactive in doing that.” (PG_HP_001) “I think it’s [the health check] something that would be very helpful for autistic people, in a sense of … I put off a lot of medical stuff because it’s uncomfortable. I feel that something like this, even if it was once a year or something like that, like health check-up, covering all bases and looking at all health overall. It’s just done then and it’s not something you need to think about.” (PN_AA_013) “I mean perhaps it ought to be explained a bit better to people that a health check does not mean a thorough physical examination by a doctor …” (PO_AA_001) “So, it was just that going into a health appointment where somebody knew that about me, I just found really helpful and I was more relaxed …” (PU_AA_001) “To be honest, I thought it would end up being similar to other times I visit the GP where it is just that in, out because they need to see the next patient in 20 minutes. But it was a lot calmer, like it was a lot more – I would say informal, but it was also formal, if that … it’s more relaxed, it wasn’t just information, information.” (PA_AA_005) “Just having that dedicated space to be like this is what’s going on with my health at the minute, these are my concerns, this is what’s going well. It was really good to have that time where I could just sort of sit and think about what’s going on with me? What do I need to bring up? It was good. It was helpful.” (PA_AA_014)
**Cognitive Participation** *Engaging autistic people and primary care staff to support health checks for autistic people*		
	• Some autistic people were highly engaged with all aspects of the health check, including completing the PAQ. • Others declined or struggled with completing the PAQ and did not all agree on how helpful the PAQ was to complete prior to the appointment. • Autistic people reported anxiety about the unknown aspects of the appointment. • Pre-appointment materials (leaflets, PAQs) helped with autistic people’s preparation. • Having the health check in the right setting, with clinicians who were perceived to be approachable, and understanding was crucial. • Recognition of the requested reasonable adjustments, in particular communication preferences, supported autistic adult engagement in the appointment. • Carers/supporters and autistic people reported role of carers/supporters in helping answer questions, provide emotional support, and arranging the appointment. • Autistic people reported positive experiences with the health check and saw the potential benefits to the wider autistic population. • Clinicians reported benefits of autism awareness training to facilitate positive health check appointments.	“It pretty much meant that both me and the GP were pretty much on the same page when I went in, so time wasn’t taken up at the start of going through what are my concerns and so on, so that was already taken care of before the appointment. So, when I got in, the GP was like, alright, we’ve got that your concerns are this and this and this, so let’s start discussing them.” (PA_AA_005) “I didn’t bother filling that [pre-appointment questionnaire] in … I looked at it later and it just doesn’t apply to me. I don’t have all these, like, extra needs.” (PQ_AA_005) “There was some where I was a bit kind of, I don’t know if it was just the way they were worded or something, it didn’t make a massive amount of sense to me. I couldn’t quite figure out what they were trying to ask me and I’m aware I struggle with that a little bit sometimes, like I can understand the words separately but together, I can’t figure out exactly what you’re asking.” (PE_AA_002) “So all patients that participated on the health checks for this practice, all live centrally. So they all wanted to go to the central practice but I don’t think that had been thought of, when [the practice] were going to do the study. I think the thought was, from the practice, that all patients would go to the branch on the west side of the city, and for these patients, that wasn’t really practical.” (PP_NC_001) “More the sheet at the front that had where the appointment was because further down it said what might happen … it was good to know, be completely prepared.” (PR_AA_001) “Yeah, it was very much a three-way thing, and I don’t think [autistic person’s name] would have opened up to a complete stranger to be truthful without me being there.” (PU_CS_001) “But I think quite quickly she was confident enough to answer her own questions. I think probably because we talked about her wanting to take meds for depression and anxiety and I think probably she kind of was looking for me to support her for that bit, but … So, it was kind of like – yeah, so I think I supported her.” (PQ_CS_001) “One person came with a supporter, everyone else came on their own. The one person who came with the supporter was the person who had a slightly greater degree of health needs and things like that, so their sister came with them. As I say, they are someone who would normally I think would have struggled were they on their own, they were often deferring to their sister, but that was able to be very helpful.” (PI_HP_001) “No, I just think for both of us it was quite a positive session sort of thing for us both, it was, because I get concerned and a little anxious of how [autistic person’s name] is going to be in those situations, and I can only say that they were very good so I think that it was a very positive two hours definitely.” (PO_CS_001) “So, it was just that going into a health appointment where somebody knew that about me, I just found really helpful, and I was more relaxed than I would have been if I had the anxiety around should I declare that? Do I need to declare that? So, it was nice going in and somebody having that awareness about me and being aware that I might need some minor adjustments … there might be some differences and other things they may have seen that day.” (PU_AA_001) “Yes, it was helpful and it to refocus on the needs of that particular group of patients. Yes, and I think it should be ongoing for all staff, from reception staff right the way through to clinicians. I think it should be something that is offered and developed.” (PW_HP_001) “With no prior knowledge, any sort of training or assistance was really useful because I didn’t really know what to expect. Again, just having that insight into autism in general and adjustments and yeah, it allowed me to make … I considered it a lot more than I probably would have done before. So, it was really good. It was useful.” (PO_HP_001)
**Collective Action** *The work done to deliver the health check in practice*		
	• The PAQ helped structure the appointment and highlight key topics to focus on for the autistic adult. • Clinicians reported mixed responses in getting the PAQ back from autistic people to be able to book appointments. • Clinicians used different approaches to arranging the appointments, some blocking out clinics, other offering ad hoc appointments. • Different models of delivery used – GP-led, nurse-led, Health Care Assistant and GP. • Appointments generally took 45–60 minutes. For some of the appointments it was challenging to fit in everything the patient wished to discuss within that time slot and a further follow-up appointment had to be made. • Clinicians reported most reasonable adjustments were simple to put in place. • Some adjustments could not be put in place due to restraints within the study context (e.g. request for female clinician, but only male GP trained). • Autistic people mostly found the health check appointment to be a positive experience, facilitated by the clinician’s manner and communication through the appointment. • Clinicians reported using clinical judgement to work through the relevant sections of the health check.	“They filled out the pre-appointment questionnaire which was hugely insightful for me because I’ve not had much interaction or sort of healthcare with autistic people. So for me that was really useful and I think for them it allowed them to get their thoughts across without being under pressure and in the moment and putting them on the spot so I thought that was good for them. It allowed me to plan how I could best conduct the health check to make the most of the time that we had together and achieve sort of optimal outcomes.” (PO_HP_001) “It was just trying to get them all in, fit them all in, get them in on first come, first served, who got them back first and try and get them in as soon as possible but it was hard when they all come back together, and we just didn’t have that many appointments for that many coming back.” (PA_NC_001) “I’m very much, and I appreciate I know you have a nurse, and a GP led model but I’m very much a, and these are individuals with generally complex unmet needs who are presenting with undifferentiated illnesses which present in more complex ways that the rest of the population. I really feel this is GP bread and butter. And I think it sits with GPs to devote the time to do this. I know my nurse colleagues are usually more procedural led and I feel like these checks, as with the, and I think this with learning disability checks as well should be the remit of GPs.” (PA_HP_002) “I spent, I can’t remember how long but must have been around 30 minutes completing that and my understanding is that it was really helpful when I met with the pharmacist because they already had quite a lot of information so that was good. And I think for me, then going into the face-to-face meeting with the [clinician] was reassuring that they had that information so there were certain things we didn’t have to go through at that time and we could focus on other things like the physical health checks.” (PU_AA_001) “Two very different patients. Yeah. One was an hour long and one was half an hour long. In terms of the timing, it very much depends on the patient you have at the time.” (PW_HP_001) “She made sure the lights weren’t as bright so I could concentrate a little bit better and that was really positive.” (PE_AA_001) “There was no dramatic changes [adjustments] that had to be made and nobody requested that. As I say, save and except some of them just needed a little bit more time on answering questions and stuff like that which you get anyway, I suspect.” (PJ_NC_001) “Because obviously I’m familiar with the surgery, so usually if I need an adjustment, I’ll accommodate that myself.” (PA_AA_014) “We did only have one female GP carrying out the health checks. Some of the patients did request a female GP. I did have to ring them and say, it is going to be a male unfortunately, we cannot get that reasonable adjustment in place, but they were all fine with it.” (PA_NC_001) “I think it [pre-appointment questionnaire] was slightly heavy [on reasonable adjustments] and I do kind of worry about you know kind of, “Well you’ve not met my reasonable adjustments so you’re not going to be able to,” how does that frame the consultation? “You’ve not done this, well you’re not going to be able to help with x, y and z.” (PA_HP_002) “I felt the nurse I saw was interested which again, it’s not always the case when you go for appointments that they don’t always seem interested. And it was, everything was explained properly which always helps. I was able to, I wasn’t getting confused or anything like that.” (PF_AA_002) “I also used the approach that I might use in a learning disability review as well. That kind of template-driven longer physical health check so that process was familiar to me from doing learning disability reviews in the practice as well. I approached it in a similar way and tried to marry all of that with the patient’s expectations and what they wanted to get from the appointment as well.” (PV_HP_001) “I didn’t do everything all of the time on the template, I just … and a bit like we do with other health checks, pick out what’s relevant to the person sat in front of you and you ask the questions that are relevant to the person sat in front of you, so it doesn’t sound too robotic. But as a prompt, it was really useful.” (PM_HP_001)
**Reflexive Monitoring** *The knowledge work people do to build accountability and maintain confidence in a set of practices*		
	• Autistic people reported a positive outcome from the health check appointment, including referrals for further tests, reassurance regarding health status, and the provision of health promotion. • Carers/supporters gained more understanding of the autistic people and saw them coping well. • Clinicians saw benefits of longer appointment and time to understand autistic people needs. • Autistic people reported increased confidence in accessing primary care and clinicians increased awareness of what can be done to support the autistic adult attending. • Autistic people highlighted future roll out would need to continue autism awareness training and consistent use of reasonable adjustments to maintain uptake. • Clinicians raised concerns about current time pressures in primary care to provide in-depth health checks. • Clinicians noted the health check would need to be adequately funded and incentivised to allow for appropriate clinical time.	“It was really useful. I’ve got an [test] booked in and I’ve had extra blood tests and things done.” (PA_AA_014) “So a lot of them I’ve managed to signpost to, kind of, health and wellbeing coaches. So diet, exercise, weight seems to be a big issue with the majority of these patients so I’ve signposted them for further advice.” (PJ_HP_002) “It was reassurance mostly that everything’s in order, my health’s fine. I’ve got nothing to be worried about.” (PB_AA_003) “In essence, if [autistic person]’s being helped then of course it helps me because it makes it easier for me to get them there. It makes it easier for the whole process.” (PU_CS_001) “But it did give chance to, kind of, unpick some issues that may have not been addressed otherwise and, kind of, cover some health issues that they may have not picked up on or we might not have picked up on, or it could have been six months down the line when they attended for an asthma review, for example, so it was one way of, kind of, seeing that patient and addressing issues.” (PJ_HP_002) “There was somebody I spoke to, I can’t remember, who said, ‘I didn’t realise, I thought when all the appointments have gone, that was it.’ I was like, no, it’s it for routine non-urgent problems, but if you are acutely unwell on the day, we will see you. And then, ‘Oh, I didn’t realise,’ was like, yeah. And perhaps it’s just understanding how the system works.” (PM_HP_001) “I think yeah, definitely I think, it’s a value and I think it has allowed us to hopefully to build those relationships, that relation connected to primary care that we, it so often gets lost when things are busy. The problem is this, the outcome is this, bye. Whereas I think as a relational thing, which is so important to then deal with the things as they pop up later on down the line, I think it’s hugely important.” (PA_HP_002) “Well I suppose the, as with all things in GP it’s time and money isn’t it now? Offering an in-depth kind of health check with someone that takes an hour … we have to submit how many appointments we offer … we do get penalised for these things.” (PG_HP_001)

AA: autistic adult; CS: carer/supporter; HP: clinician; NC: non-clinical staff.

ID: [Practice Code_Participant type_participant number].

#### “Coherence” (Work Done to Understand and Plan the Activities That Need to Be Accomplished to Put an Intervention and its Components Into Practice)

There was collective agreement across autistic people, carers/supporters, and clinicians on the overarching purpose and value of health checks for autistic people. There was evidence that coherence needs to be improved. Some autistic people described the health check as more comprehensive than expected and others were expecting more from it than they received. This may be down to different experiences of the health check appointment across different primary care practices but also indicates that future implementation needs to consider managing autistic people’s expectations of the health check appointment to improve coherence.

Clinician views differed in their descriptions of who the health check should be for, for example, whether “younger, fit, healthy, early 20s” should be invited for the health check. While they could still see how this group could benefit from the health check and there is value in bringing people in for a review, some clinicians struggled to see how time could be allocated within primary care to provide reviews for this group.

There was a recognition from autistic people and clinicians that the health check was a different offer to usual care for autistic people, with the focus on autism awareness, improving accessibility and providing a longer appointment. Across all the stakeholder groups, there was agreement that the health check was suitable and needed for autistic people.

#### “Cognitive Participation” (Relational Work That People Do to Build and Sustain a Practice/Intervention)

To successfully deliver the health check, engagement in all its constituent parts (clinician training, PAQ, appointment, and action plan) is needed. Clinicians and the non-clinical staff reported challenges in receiving the PAQ from the autistic people ahead of the health check which created delays in booking the health check appointment. Engagement with the health check processes varied among autistic people. While some were highly engaged, completing the PAQ and attending the health check appointment, others struggled with some aspects of it. A small number of autistic people did not see any benefit in completing the PAQ so did not engage with it, while others commented that the questionnaire was lengthy and difficult to complete due to uncertainty about how to answer some questions, leading to stress for some people.

Factors that facilitated engagement included pre-appointment materials (information leaflets), the use of reasonable adjustments (e.g. quiet waiting areas, adjusted lighting), and support from carers. Carers/supporters helped autistic people with completing the PAQ and attending the health check. Each group highlighted that carer/supporter involvement facilitated communication in the health check appointment and provided emotional support to autistic people when needed during and after the health check appointment.

Overall, engagement with the health check was reported to be good and most of the autistic people reported it to be a positive experience and better than previous primary care experiences. With the specifically designed health check, autistic people reported feeling more comfortable, in control and listened to, compared to previous experiences with primary care and other healthcare settings. Equally, clinicians had a positive experience of delivering the health check and recognised that it was an opportunity to spend longer with patients and see those they would not normally see.

#### “Collective Action” (Operational Factors Needed to Deliver the Health Check)

Delivering the health check involved reviewing the PAQ, arranging the appointment, implementing reasonable adjustments, delivering the health check and creating a health action plan. Where PAQs were returned both clinicians and autistic people reported this was helpful in preparing for and structuring the health check. Clinicians adopted different approaches to appointment scheduling, with some blocking hours of time for multiple health checks while others offered ad hoc slots. For autistic people, it was important that their preferred contact method was used when arranging the health check appointment. The length of appointments (typically 45–60 minutes) was generally considered appropriate, although some patients required longer appointments or additional follow-ups depending on what they presented with.

Reasonable adjustments were implemented where possible, though not all requests (e.g. clinician gender preferences) could be accommodated. Some clinicians also raised concerns about the number of reasonable adjustments listed in the PAQ and how to manage patients’ expectations of what can and cannot be put in place and the impact this may have on consultations.

When delivering the health check, some clinicians reported using clinical judgement on which sections of the clinician health check template were most relevant to cover and found the process similar to how they would carry out other health checks.

#### “Reflexive Monitoring” (Participants’ Appraisal of the Worth and Effectiveness of the Health Check and How They Thought it Could Be Implemented Into Future Primary Care)

All autistic people reported a positive outcome from the health check appointment, including referrals for further tests, reassurance regarding health status, and the provision of health promotion. Carers/supporters shared that anything that supported the autistic adult was a positive outcome for themselves as well, and some discussed gaining a better understanding of the autistic people as well. Similarly, clinicians saw the benefits of the health check for the autistic adult and also recognised the benefits of having a longer appointment and getting to know the autistic adult and their needs better.

When considering the future delivery of the health check within the NHS and other health systems (outside the context of the research trial), autistic people raised that there would need to be increased autism awareness from all staff in primary care and the consistent use of reasonable adjustments to remove some of the barriers to accessing primary care. Some autistic people discussed that future engagement in health checks would require autistic people receiving clear information about what the appointment was for and what would be covered to encourage attendance.

Clinicians reflected that work would be needed to encourage engagement in the health checks by the autistic adult population. To implement the health check as part of a regular offer within primary care, clinicians raised there would need to be adequate funding and incentivisation and suggested following a similar model to the NHS health checks for people with an intellectual disability currently offered, in terms of inviting people by birth month and having the funding to back-fill clinical time.

Overall, most participants interviewed recognised that the health checks delivered in the trial had been beneficial for all stakeholder groups, and that the health check should be offered to autistic people in NHS primary care in the future.

## Discussion

This study evaluated the acceptability and factors related to implementation of a tailored health check for autistic people in primary care, using a mixed-methods approach. Our findings demonstrate ways in which the health check could be implemented in an acceptable and beneficial way within local services. The health check was acceptable to autistic people, carers/supporters, and clinicians, with participants reporting completion of all the health check components and identifying clear benefits for engagement with healthcare services and identification of health and social needs. These findings build on earlier stages of this research (Mason et al., 2021; Taylor et al., 2023) and prior findings that interventions tailored to autistic needs can improve engagement and address unmet needs (Nicolaidis et al., 2016; Rodgers et al., 2024). Some implementation challenges were identified with implications for future implementation at scale.

NPT analysis provided a nuanced understanding of the processes required to embed health checks into routine primary care practice for autistic people. Participants’ understanding (coherence) of the health check was generally strong. Autistic people, carers/supporters, and clinicians recognised the health check as an opportunity to address health inequalities and improve accessibility. Disparities in understanding between autistic people and clinicians suggest the need for clear, consistent communication about its purpose, structure, and recipients. Findings around clinicians’ coherence align with previous research indicating the need to increase education among primary care staff of the barriers autistic people face in accessing healthcare and increase their knowledge of autism (Mason et al., 2022; Walsh et al., 2023; Warreman et al., 2024).

Engagement (cognitive participation) in the health check was evident but varied. Questionnaires and interviews showed variation in autistic people’s engagement with the PAQ. Many found the PAQ helpful, reflecting earlier research on preparation and reasonable adjustments (Mason et al., 2022; Taylor et al., 2023). However, the mixed engagement underscores the heterogeneity of the autistic community (Nicolaidis et al., 2014) and the need for flexibility in reporting barriers to accessing healthcare and adjustments needed.

Delivery of the health check was feasible within existing primary care structures. Clinicians used the PAQ to tailor appointments, implement reasonable adjustments as requested, and provided health action plans. Barriers were consistent with prior studies, including administrative burdens and inconsistent adjustment implementation as noted in the AASPIRE Healthcare Toolkit studies in the United States (Kang et al., 2022; Nicolaidis et al., 2016), and also in the implementation of CHAP in Australia (Lennox et al., 2016). These studies highlight the need for streamlined processes, adequate resources, and flexibility to integrate health checks into routine practice.

Appraisal of the intervention was overwhelmingly positive, echoing previous research showing longer, autism-aware appointments can improve patient comfort, facilitate discussion and support care (Nicolaidis et al., 2020; Saqr et al., 2018). However, clinicians raised concerns about sustaining the health check without protected time or additional funding. Similar to Davies et al. (2024), while primary care staff saw the benefits and value of the health check, concerns were raised about how to resource the health check delivery, in terms of financial incentives as well as clinical capacity. Clinicians suggested aligning the health check for autistic people with existing incentivised frameworks (e.g. NHS health checks for people with a Learning Disability) to secure clinician engagement and ensure equitable delivery.

Together, these findings show that the co-designed health check for autistic people is acceptable, and successful implementation will require some system-level adaptations: consistent patient communication, autism-specific training for all staff, securing administrative and clinical support, and integration into incentivised care pathways.

### Strengths and Limitations

This study was carried out within the UK NHS, where provision is free for patients at the point of care. Practices in the trial received funding to deliver the health check, thereby providing the opportunity to gather information on the feasibility of delivering the health check in this context and factors important in terms of implementation. The high response rates to the evaluation questionnaires among autistic people (85%) and clinicians (75%) enhance the robustness of the findings. The purposive sampling strategy for interviews allowed a diversity of autistic adult experiences to be captured, providing insights into both barriers and facilitators to implementation. The experiences of carers/supporters were less well-represented due to lower recruitment rates. Similarly, recruitment of non-clinical staff was lower than expected, meaning further understanding around the processes of sending out the PAQ and arranging the health check appointment could not be collected. Future research could explore these perspectives further in the context of implementation. Finally, the health check delivery was conducted within a research context with additional requirements and support structures, and further research will be helpful to understand implementation under a routine service context. It should also be noted that, due to resource limitations, study materials and questionnaires were only available in English, without provision for British Sign Language (BSL) or other language interpretation. This may have impacted the inclusivity of the study and the representativeness of the findings.

## Conclusion

This evaluation demonstrates that a tailored health check for autistic people is acceptable in primary care settings, with benefits reported by autistic people, carers/supporters, and clinicians alike. The health check was designed to be used across the whole adult age and intellectual ability range and is an important opportunity to identify unmet health needs, promote preventive care, and improve engagement with primary healthcare services. These priorities align closely with the *NHS 10-Year Health Plan for England* (National Health Service England, 2025), which commits to reducing health inequalities, expanding personalised and preventive care, using technology, and improving community-based services for underserved populations, including autistic people through neighbourhood healthcare delivery approaches.

Results of the clinical and cost-effectiveness outcomes from this trial may influence whether health checks are provided within the NHS and other health systems and will be presented in a forthcoming article, which is in preparation. To deliver health checks effectively and sustainably, primary care will require investment from primary care practices in taking part in enhanced administrative support, and adequate resourcing, including incentives to protect clinical time. Ensuring consistency in reasonable adjustments and preparing both patients and staff will be essential to maximise uptake and effectiveness. With thoughtful and supportive implementation, tailored health checks have the potential to reduce health inequalities, improve patient outcomes including reducing early morbidity and avoidable mortality, and transform primary care experiences for autistic people.

## Supplemental Material

sj-docx-1-aut-10.1177_13623613261433106 – Supplemental material for Acceptability and Implementation of a Primary Care Health Check for Autistic People: Findings From Evaluation Questionnaires and Interviews Supplemental material, sj-docx-1-aut-10.1177_13623613261433106 for Acceptability and Implementation of a Primary Care Health Check for Autistic People: Findings From Evaluation Questionnaires and Interviews by Hannah Merrick, Helen Taylor, Barry Ingham, Tracy Finch, Sarah Al-Ashmori, Ruby Herrington, Clare Scarlett, Carole Buckley, Sally-Ann Cooper, Cristina Fernandez-Garcia, Shona Haining, Rhianna Lees, Nicholas Lennox, Sebastian Moss, Tim Nicholls, Christina Nicolaidis, Malcolm Osborne, Dora M Raymaker, Tomos Robinson, Anna Urbanowicz, James MS Wason, Colin Wilson and Jeremy R Parr in Autism

sj-docx-2-aut-10.1177_13623613261433106 – Supplemental material for Acceptability and Implementation of a Primary Care Health Check for Autistic People: Findings From Evaluation Questionnaires and Interviews Supplemental material, sj-docx-2-aut-10.1177_13623613261433106 for Acceptability and Implementation of a Primary Care Health Check for Autistic People: Findings From Evaluation Questionnaires and Interviews by Hannah Merrick, Helen Taylor, Barry Ingham, Tracy Finch, Sarah Al-Ashmori, Ruby Herrington, Clare Scarlett, Carole Buckley, Sally-Ann Cooper, Cristina Fernandez-Garcia, Shona Haining, Rhianna Lees, Nicholas Lennox, Sebastian Moss, Tim Nicholls, Christina Nicolaidis, Malcolm Osborne, Dora M Raymaker, Tomos Robinson, Anna Urbanowicz, James MS Wason, Colin Wilson and Jeremy R Parr in Autism

sj-docx-3-aut-10.1177_13623613261433106 – Supplemental material for Acceptability and Implementation of a Primary Care Health Check for Autistic People: Findings From Evaluation Questionnaires and Interviews Supplemental material, sj-docx-3-aut-10.1177_13623613261433106 for Acceptability and Implementation of a Primary Care Health Check for Autistic People: Findings From Evaluation Questionnaires and Interviews by Hannah Merrick, Helen Taylor, Barry Ingham, Tracy Finch, Sarah Al-Ashmori, Ruby Herrington, Clare Scarlett, Carole Buckley, Sally-Ann Cooper, Cristina Fernandez-Garcia, Shona Haining, Rhianna Lees, Nicholas Lennox, Sebastian Moss, Tim Nicholls, Christina Nicolaidis, Malcolm Osborne, Dora M Raymaker, Tomos Robinson, Anna Urbanowicz, James MS Wason, Colin Wilson and Jeremy R Parr in Autism

sj-pdf-4-aut-10.1177_13623613261433106 – Supplemental material for Acceptability and Implementation of a Primary Care Health Check for Autistic People: Findings From Evaluation Questionnaires and Interviews Supplemental material, sj-pdf-4-aut-10.1177_13623613261433106 for Acceptability and Implementation of a Primary Care Health Check for Autistic People: Findings From Evaluation Questionnaires and Interviews by Hannah Merrick, Helen Taylor, Barry Ingham, Tracy Finch, Sarah Al-Ashmori, Ruby Herrington, Clare Scarlett, Carole Buckley, Sally-Ann Cooper, Cristina Fernandez-Garcia, Shona Haining, Rhianna Lees, Nicholas Lennox, Sebastian Moss, Tim Nicholls, Christina Nicolaidis, Malcolm Osborne, Dora M Raymaker, Tomos Robinson, Anna Urbanowicz, James MS Wason, Colin Wilson and Jeremy R Parr in Autism
