# Supplementary figures and images for: Acceptability and Implementation of a Primary Care Health Check for Autistic People: Findings From Evaluation Questionnaires and Interviews
Source: Autism. 2026 Jun 16;30(8):1955–70. doi: 10.1177/13623613261433106 (PMC13392152; doi:10.1177/13623613261433106)

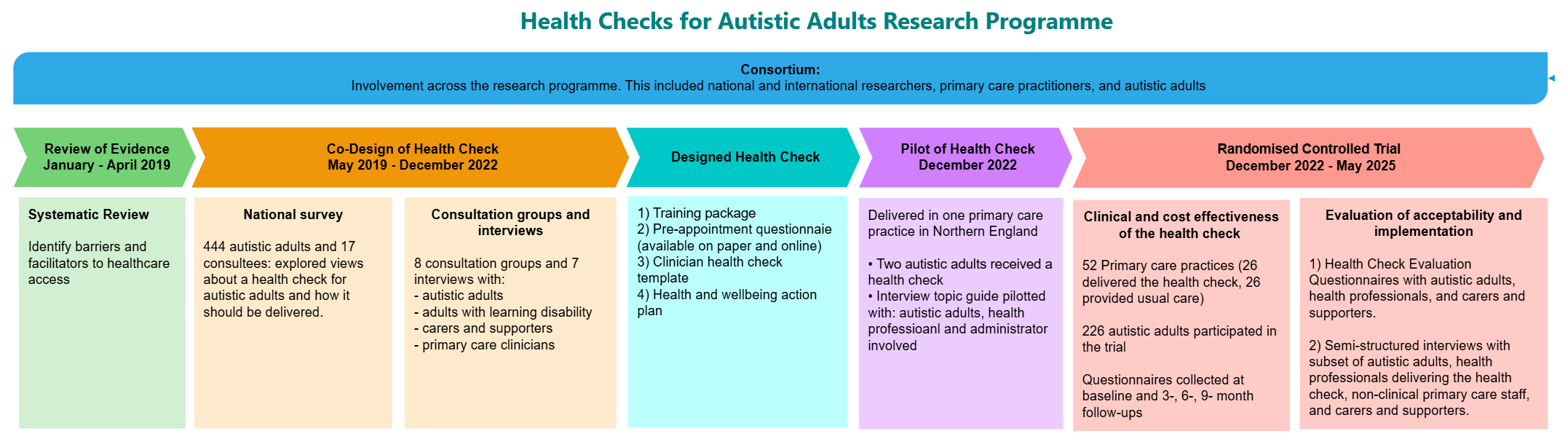

Supplement: sj-docx-1-aut-10.1177_13623613261433106 – Supplemental material for Acceptability and Implementation of a Primary Care Health Check for Autistic People: Findings From Evaluation Questionnaires and Interviews [file sj-docx-1-aut-10.1177_13623613261433106.docx]
