# Supplementary material for: Acceptability and Implementation of a Primary Care Health Check for Autistic People: Findings From Evaluation Questionnaires and Interviews
Source: Autism. 2026 Jun 16;30(8):1955–70. doi: 10.1177/13623613261433106 (PMC13392152; doi:10.1177/13623613261433106)
Supplement: sj-docx-2-aut-10.1177_13623613261433106 – Supplemental material for Acceptability and Implementation of a Primary Care Health Check for Autistic People: Findings From Evaluation Questionnaires and Interviews [file sj-docx-2-aut-10.1177_13623613261433106.docx]

**Supplementary File 1: Interview Topic Guides**

**Health Checks for Autistic Adults Study**

**Autistic Adults, Carers and Supporters Interview Topic Guide**

*Note: The interview schedule is developmental. The questions will need to be tailored to the discussion in the interview. The schedule given here is therefore a general topic guide and additional questions may be asked if indicated.*

The evaluation questionnaires have given us lots of information about what autistic adults, carers and relatives thought about the health check. I’d like to talk about some of the results and discuss these in more detail with you. We particularly want to focus on how useful the health check is and any changes needed to make it used widely in usual clinical care.

**1. Thinking back, what was your experience of completing the health check pre-appointment questionnaire (sent to you by your GP practice before the health check appointment)?**

*Show blank version of health check pre-appointment questionnaire to participant to help them remember.*

*Additional prompts to use as needed:*

- *How helpful was it to have time to think about the information being shared prior to the health check appointment?*
- *How useful was the Pre-Appointment Questionnaire in your own preparation for the health check appointment itself?*
- *From evaluation questionnaire, know it took X minutes to complete – did that feel an ok amount of time? If wanted shorted, what would take out of it?*
- *If needed support, explore what the support was and what needed with*
- *Ask any relevant questions related to their HCEQ*

**2. Thinking back, what was your experience of attending the health check appointment?**

*Additional prompts to use as needed:*

- *How did it compare to what you expected?*
- *Did you come on your own to the appointment?*
- *How did the appointment go overall?*
- *Did the clinician use the PAQ you completed during the appointment?*
- *Are there any positive or negative aspects that you would like to highlight?*

**3.What’s your main impression of how useful the health check is?**

*Additional prompts to use as needed:*

*Health check pre-appointment questionnaire:*

- *Did it help you / the autistic adult effectively share your / their communication needs? In what way/how?*
- *Was the lists of adjustments useful? In what way/how?*
- *How did you feel about the questions asked about your / their health and wellbeing?*

*Health check appointment:*

- *Was the health check appointment useful?*
- *Were the adjustments you / they requested in place?*
- *Did the health check address health or other concerns you had?*
- *Did the health check pick up any new health conditions or issues?*
- *Was it reassuring to have had the health check?*

**4. [If a carer/supporter attended the appointment] – Have you seen any impacts of the health check for [autistic adult name] on your own health or quality life?**

- *Has it changed anything about your role in supporting or caring for [autistic adult]?*
- *Have there been any positives/benefits of [autistic adult] participating in the health check for yourself?*
- *What positive impacts do you think the health check has had on [autistic adults] quality of life and health?*

**5. Can you think of anything about the health check pre-appointment questionnaire or health check appointment that definitely needs to be improved?**

**Health Checks for Autistic Adults Study**

**General Practice Staff Interview Topic Guide**

*Note: The interview schedule is developmental. The questions will need to be tailored to the discussion in the interview and the individual’s level of experience/expertise. The schedule given here is therefore a general topic guide and additional questions may be asked if indicated.*

The evaluation questionnaires have given us lots of information about what health professionals thought about the health check. We particularly want to focus on how useful the health check is and any changes needed to make it used widely in usual clinical care. When we talk about the health check, we are referring to the pre-appointment questionnaire (that was completed by the patient beforehand) and the health check appointment itself.

1. **a) Thinking of the health check appointments you’ve undertaken, how do you feel they have gone?**

*Additional prompts to use as needed:*

- - *How did you use the PAQ during the appointment?*
  - *Was it helpful reviewing that prior to the appointment?*
  - *How did you find filling in the Health Action Plan?*
  - *How helpful was the training in preparing you for and delivering the health check appointment? i.e. autism awareness training.*

**b) How have you experienced the engagement of patients in this process?**

*Additional prompts to use as needed:*

- *How variable was the patient(s) engagement?*
- *Did the patient(s) come on their own to the appointment?*
- *Did the patient(s) request any reasonable adjustments? If so, how easy was it to put these into place?*

**2. What is your main impression of how useful the health check is?**

*Additional prompts to use as needed:*

- *How useful was the health check appointment for the patient? How useful was the health check appointment for you supporting the patient?*
- *Were staff able to put into place the adjustments requested?*
- *How did the health check affect the professional patient interaction?*
- *Did the health check pick up any new health conditions or issues?*
- *Did any aspects of the health check lead to or cause difficulties* ***for the patient?***
- *Did any aspects of the health check lead to or cause difficulties* ***for yourself?***

1. **Can you think of anything about the health check that definitely needs to be improved?**

*Additional prompts to use as needed:*

- *Pre-appointment questionnaire procedure?*
- *Arranging the health check?*
- *Content of the health check template?*
- *Changes to the health check action plan?*

**3. What barriers might be a challenge to wider use of the health check in NHS primary care? What needs to be in place for the health check to be widely used?**

*Additional prompts to use as needed:*

- *How could those barriers/challenges be addressed?*
- *How would you implement the health check in your practice?*
- *How would you get your colleagues to use the health check?*

**Health Checks for Autistic Adults Study**

**Non-clinical General Practice Staff Interview Topic Guide**

*Note: The interview schedule is developmental. The questions will need to be tailored to the discussion in the interview and the individual’s level of experience/expertise. The schedule given here is therefore a general topic guide and additional questions may be asked if indicated.*

When we talk about the health check, we are referring to the pre-appointment questionnaire (that was completed by the patient beforehand) and the health check appointment itself.

1. **a) Thinking of the health check appointments you’ve arranged, how do you feel that process has gone?**

**b) How have you experienced the engagement of patients in this process?**

*Additional prompts to use as needed:*

- *How was the process of sending out the PAQs and getting them returned? Anything that could be done to improve this process?*
- *How variable was patient engagment?*
- *Did the patients come on their own to the appointment?*

1. **Were you responsible for arranging any reasonable adjustments requested? If so, how did you find this process?**

*Additional prompts to use as needed:*

- *What were the challenges around arranging reasonable adjustments?*
- *Do you forsee any challenges in implementing reasonable adjustments long term?*
- *Were there any benefits/positives in asking about the reasonable adjustments?*

1. **Can you think of anything about the health check that definitely needs to be improved?**

*Additional prompts to use as needed:*

- *Pre-appointment questionnaire procedure?*
- *Arranging the health check?*

**3. What barriers might be a challenge to wider use of the health check in NHS primary care? What needs to be in place for the health check to be widely used?**

*Additional prompts to use as needed:*

- *How could those barriers/challenges be addressed?*
- *How would you implement the health check in your practice?*
- *How would you get your colleagues to use the health check?*
