# Supplementary material for: Acceptability and Implementation of a Primary Care Health Check for Autistic People: Findings From Evaluation Questionnaires and Interviews
Source: Autism. 2026 Jun 16;30(8):1955–70. doi: 10.1177/13623613261433106 (PMC13392152; doi:10.1177/13623613261433106)
Supplement: sj-docx-3-aut-10.1177_13623613261433106 – Supplemental material for Acceptability and Implementation of a Primary Care Health Check for Autistic People: Findings From Evaluation Questionnaires and Interviews [file sj-docx-3-aut-10.1177_13623613261433106.docx]

**Supplementary File 1**

*Table S1. Autistic adult’s additional demographic data*

|  | *Responder* |
| --- | --- |
|  | *N = 81 (%)* |
| *Numbers with demographic data available* | *N = 80 (99%)* |
| *Age diagnosed (years)* |  |
|  |  |
| *Mean (SD)* | *26.9 (16.5)* |
| *Median (IQR); Range* | *22.0 (16.0, 38.5); 2.0, 63.0* |
| *Numbers with data* | *76 (95%)* |
| *Highest level of qualification* | *N= 80 (99%)* |
| *No formal* | *7 (8.8%)* |
| *Basic Skills* | *3 (3.8%)* |
| *GCSE* | *13 (16.2%)* |
| *A Levels* | *20 (25.0%)* |
| *Certificate of higher education* | *4 (5.0%)* |
| *Diploma of higher education* | *2 (2.5%)* |
| *Bachelor’s degree* | *17 (21.2%)* |
| *Post graduate degree* | *14 (17.5%)* |
| *Health Conditions and Status* | *N= 80 (99%)* |
| *Difficulties with learning diagnosed^3^* |  |
| *None* | *53 (66.2%)* |
| *1-2 reported* | *24 (30.0%)* |
| *3-4 Reported* | *3 (3.8%)* |
| *Difficulties with learning suspected^3^* |  |
| *None* | *46 (57.5%)* |
| *1-2 reported* | *31 (38.8%)* |
| *3-4 Reported* | *3 (3.8%)* |
| *Mental health or neurological conditions diagnosed^3^* |  |
| *None* | *21 (26.2%)* |
| *1-2 reported* | *45 (56.2%)* |
| *3-4 Reported* | *11 (13.8%)* |
| *5 or more* | *3 (3.8%)* |
| *Mental health or neurological conditions suspected^3^* |  |
| *None* | *28 (35.0%)* |
| *1-2 reported* | *40 (50.0%)* |
| *3-4 Reported* | *12 (15.0%)* |
| *Mental health treatment* |  |
| *Numbers receiving treatment* | *37 (46.2%)* |
| *Type of treatment received:* |  |
| *Medication* | *35 (94.6%)* |
| *Therapy* | *9 (24.3%)* |
| *Currently on waitlist* | *3 (8.1%)* |
| *Physical health conditions diagnosed^3^* |  |
| *None* | *25 (31.2%)* |
| *1-2 reported* | *31 (38.8%)* |
| *3-4 Reported* | *13 (16.2%)* |
| *5 or more* | *11 (13.8%)* |
| *Physical health conditions suspected^3^* |  |
| *None* | *36 (45.0%)* |
| *1-2 reported* | *31 (38.8%)* |
| *3-4 Reported* | *10 (12.5%)* |
| *5 or more* | *3 (3.8%)* |
| *Physical health treatment* |  |
| *Numbers receiving treatment* | *43 (53.8%)* |
| *Type of treatment received:* |  |
| *Medication* | *41 (95.3%)* |
| *Therapy* | *7 (16.3%)* |
| *Waitlist* | *2 (4.7%)* |
| *Support needs* | *N= 80 (99%)* |
| *Numbers receiving support currently* | *53 (66.2%)* |
| *Support received in:* |  |
| *In the home* | *32 (60.4%)* |
| *Employment* | *16 (30.2%)* |
| *Health care* | *27 (50.9%)* |
| *Finance* | *34 (64.2%)* |
| *Social activities* | *23 (43.4%)* |
| *Lifelong learning activities* | *14 (26.4%)* |
| *In the community* | *20 (37.7%)* |
| *Organisation* | *20 (37.7%)* |
| *Other* | *1 (1.9%)* |
| *Who do you receive support from:* |  |
| *Family member* | *40 (75.5%)* |
| *Friend* | *9 (17.0%)* |
| *Advocate* | *1 (1.9%)* |
| *Support care worker* | *5 (9.4%)* |
| *Professional* | *6 (11.3%)* |
| *Support group(s)* | *3 (5.7%)* |
| *Colleague at work* | *10 (18.9%)* |
| *Religious organisation* | *1 (1.9%)* |
| *University* | *1 (1.9%)* |
| *Government benefits* | *1 (1.9%)* |
| *Access to work* | *0 (0.0%)* |
| *Other professional* | *3 (5.7%)* |
| *Area’s of support wanted* | *N= 80 (99%)* |
| *Numbers wanting support* | *58 (72.5%)* |
| *In the home* | *15 (25.9%)* |
| *Employment* | *20 (34.5%)* |
| *Health care* | *24 (41.4%)* |
| *Finance* | *25 (43.1%)* |
| *Social activities* | *33 (56.9%)* |
| *Lifelong learning activities* | *20 (34.5%)* |
| *In the community* | *8 (13.8%)* |
| *Organisation* | *11 (19.0%)* |
| *Other* | *3 (5.2%)* |
| *Social Communication Difficulties* | *N= 78 (96%)* |
| *SRS scores by severity category:* |  |
| *Normal* | *4 (5.1%)* |
| *Mild* | *3 (3.8%)* |
| *Moderate* | *26 (33.3%)* |
| *Severe* | *45 (57.7%)* |
| *Mean (SD)* | *76.7 (9.4)* |
| *Median (IQR); Range* | *77.0 (72.0, 82.0); 50.0, 99.0* |
| *Health Outcomes in Primary Care* |  |
| *Primary Care Outcomes Questionnaire:* |  |
| *Health and wellbeing* |  |
| *Mean (SD)* | *3.1 (1.0)* |
| *Median (IQR); Range* | *3.1 (2.4, 3.9); 1.0, 5.0* |
| *Numbers with data* | *80 (99%)* |
| *Health knowledge and self-care* |  |
| *Mean (SD)* | *3.6 (1.1)* |
| *Median (IQR); Range* | *3.8 (3.0, 4.5); 1.0, 5.0* |
| *Numbers with data* | *80 (99%)* |
| *Confidence in health provision* |  |
| *Mean (SD)* | *3.2 (1.0)* |
| *Median (IQR); Range* | *3.2 (2.7, 3.9); 1.0, 5.0* |
| *Numbers with data* | *80 (99%)* |
| *Confidence in health plan* |  |
| *Mean (SD)* | *3.0 (0.8)* |
| *Median (IQR); Range* | *3.0 (2.5, 3.5); 1.0, 4.7* |
| *Numbers with data* | *78 (98%)* |
| *Levels of independence* | *N= 80 (99%)* |
| *Waisman Activities of Daily Living Scale:* |  |
| *Mean (SD)* | *28.1 (6.4)* |
| *Median (IQR); Range* | *29.0 (25.0, 34.0); 5.0, 34.0* |

*Table S2. Carers additional demographic data*

|  | *Carers/supporters, N = 11* |
| --- | --- |
| *Employment Status** |  |
| *In education or training* | *1 (9%)* |
| *A carer / have caring responsibilities* | *5 (45%)* |
| *Employed / self-employed full time* | *3 (27%)* |
| *Employed / self-employed part time* | *3 (27%)* |
| *Unemployed and unable to work* | *1 (9%)* |
| *Retired* | *1 (9%)* |
| *Highest level of Qualification* |  |
| *No formal* | *2 (18%)* |
| *GCSE* | *2 (18%)* |
| *A-levels* | *3 (27%)* |
| *Diploma of Higher Education* | *2 (18%)* |
| *Bachelors* | *2 (18%)* |
| *How they know autistic adult* |  |
| *Parent/Grandparent of Autistic Person* | *7 (64%)* |
| *Partner* | *3 (27%)* |
| *Friend* | *1 (9%)* |
| *How long have you known autistic adults (yrs)* |  |
| *Mean (SD)* | *23.5 (8.6)* |
| *Median (IQR)* | *24.0 (21.0, 27.0)* |
| *Range* | *10.0, 43.0* |
| *How much time do you spend with them- hours per week)* |  |
| *Mean (SD)* | *85.8 (61.2)* |
| *Median (IQR)* | *63.0 (40.0, 151.0)* |
| *Range* | *0.0, 168.0* |

**Participants can select more than one option*

*Table S3. Clinicians’ additional demographic data*

|  | *Clinician, N= 18* |
| --- | --- |
| *Years in current post* |  |
| *Mean (SD)* | *10.61 (10.08)* |
| *Median (IQR)* | *7.50 (4.00, 15.00)* |
| *Range* | *0.00, 33.00* |
| *Years qualified* |  |
| *Mean (SD)* | *17.83 (12.21)* |
| *Median (IQR)* | *16.50 (8.00, 30.00)* |
| *Range* | *0.00, 37.00* |

Table S4 Autistic Adults views of the Health Check PAQ

|  | **Health Check**  *N = 81 (%)* |
| --- | --- |
| **The Health Check Pre-Appointment Questionnaire** |  |
| Was the Pre-appointment questionnaire completed before attending the health check appointment? |  |
| Yes, most, or all of it | 80 (99%) |
| No | 1 (1%) |
| Was the Pre-appointment questionnaire completed alone? |  |
| Yes | 56 (70%) |
| No, I had support | 24 (30%) |
| Type of Pre-appointment questionnaire completed? |  |
| Paper | 45 (56%) |
| Online | 33 (41%) |
| Missing | 2 (2%) |
| Was the Pre-appointment questionnaire ok to complete? |  |
| Yes | 72 (90%) |
| No | 7 (9%) |
| Can’t remember | 1 (1%) |
| Numbers indicating any difficulty with the pre-appointment questionnaire ^T^ | 33 (41%) |
| General difficulties | 25 (76%) |
| Topic specific difficulties | 22 (67%) |
| Unknown difficulty | 1 (3%) |
| General difficulties reported in open text (n=25) |  |
| Understanding/answering the question | 15 (60%) |
| Too long | 5 (20%) |
| General difficulties in completing forms/questionnaires | 1 (4%) |
| Technical difficulty with online system | 2 (8%) |
| Not inclusive | 1 (4%) |
| Anxious not to get anything wrong | 1 (4%) |
| Topic specific difficulty reported in open text (n= 22) |  |
| Upsetting to think about my problems/struggles in a concentrated format | 9 (41%) |
| Not reported | 13 (59%) |
| Was the Pre-appointment questionnaire useful? |  |
| I don’t know | 18 (22%) |
| No | 2 (2%) |
| Yes | 60 (75%) |
| If yes, what was useful about it? ^T^ |  |
| Sharing information about my communication before the appointment | 45 (58%) |
| Sharing information about the adjustments needed for the appointment | 36 (46%) |
| Sharing information about my current health concerns before the appointment | 44 (56%) |
| Sharing information about my general health and wellbeing before the appointment | 46 (59%) |
| I prefer to write things down rather than talk to others | 16 (21%) |
| It covered all the things that were important to me | 22 (28%) |
| I didn’t have to answer as many questions at the appointment | 12 (15%) |
| Guided through all health areas to avoid missing anything | 3 (4%) |
| Thinking everything through first made the appointment more efficient | 4 (5%) |
| Did the Pre-appointment questionnaire provide enough opportunities to provide detail about yourself? |  |
| Yes | 72 (90%) |
| No | 6 (8%) |
| I can’t remember | 2 (2%) |
| Time taken to complete the Pre-appointment questionnaire? |  |
| 15-30 minutes | 33 (41%) |
| 31-45 minutes | 19 (24%) |
| 46-60 minutes | 13 (16%) |
| > 1 hour | 15 (19%) |
| How did you find the time taken to complete this? |  |
| About right | 54 (68%) |
| A little too long | 19 (24%) |
| Much too long | 7 (9%) |

Table S5 Autistic Adults views of the Health Check appointment

|  | **Health check**  *N = 81 (%)* |
| --- | --- |
| **The health check appointment** |  |
| Were any adjustments requested for the health check appointment? |  |
| Yes | 37 (46%) |
| No | 44 (54%) |
| Did the GP practice contact you before your health check to confirm what adjustments would be made? |  |
| Yes | 17 (46%) |
| No | 15 (41%) |
| I requested not to be contacted | 2 (5%) |
| Unsure | 1 (3%) |
| Missing | 2 (5%) |
| Were the adjustments you requested made when you attended your health check? |  |
| Most or all of the adjustments I requested were made | 22 (59%) |
| Some of the adjustments were made | 9 (24%) |
| None of the adjustments were made | 5 (14%) |
| Missing | 1 (3%) |
| Do you think the adjustments were helpful? |  |
| Yes | 31 (84%) |
| Missing | 6 (16%) |
| If yes, how were they helpful? ^T^ |  |
| Helped me attend the appointment | 6 (19%) |
| Helped me give information to my GP/nurse | 15 (48%) |
| Helped me with waiting at my appointment | 10 (32%) |
| Helped me to remain calm and comfortable at my appointment | 22 (71%) |
| Helped me make good decisions about my health and healthcare | 13 (42%) |
| Helped make examinations, procedures or treatments more successful | 10 (32%) |
| Helped me to better understand or follow any advice | 15 (48%) |
| Felt listened to by doctor | 1 (3%) |
| Did the adjustments you received make this appointment better for you than your usual appointments at your GP practice? |  |
| Yes | 26 (70%) |
| No | 5 (14%) |
| Missing | 6 (16%) |
| Did you take anyone with you to your health check appointment? |  |
| No | 56 (69%) |
| Yes | 25 (31%) |
| If yes, did you find it helpful to have someone with you at the health check appointment? |  |
| Yes | 25 (100%) |
| No | 0 (0%) |
| Would you attend another health check appointment in the future? |  |
| Yes | 74 (91%) |
| No | 1 (1%) |
| I don’t know | 6 (7%) |
| How often do you think the health check should be offered to you? |  |
| Every year | 69 (85%) |
| Every 2-3 years | 7 (9%) |
| Every 4-5 years | 3 (4%) |
| Missing | 2 (2%) |
| Did you learn anything about your health from the health check? |  |
| Yes | 56 (69%) |
| No | 25 (31%) |

Table S6 Summary of sub-group analyses on reported evaluations

|  | **Pre-appointment questionnaire deemed useful** | | | **Attend another health check in the future** | | **Frequency of health check** | | **Learn anything from health check** | |
| --- | --- | --- | --- | --- | --- | --- | --- | --- | --- |
|  | **Age** | | | | | | | | |
|  | 40+ | <40 | | 40+ | <40 | 40+ | <40 | 40+ | <40 |
| **Yes** | 16 (94.1%) | 43 (97.7%) | | 20 (90.9%) | 53 (91.4%) | 17 (85.0%) | 51 (87.9%) | 12 (54.5%) | 43 (74.1%) |
| **No** | 1 (5.9%) | 1 (2.3%) | | 2 (9.1%) | 5 (8.6%) | 3 (15.0%) | 7 (12.1%) | 10 (45.5%) | 15 (25.9%) |
| **P value** | 0.483 | | | 1.000 | | 0.711 | | 0.110 | |
|  | **Gender** | | | | | | | | |
|  | Female | Male | | Female | Male | Female | Male | Female | Male |
| **Yes** | 27 (93.1%) | 25 (100.0%) | | 32 (88.9%) | 31 (91.2%) | 31 (88.6%) | 27 (81.8%) | 26 (72.2%) | 20 (58.8%) |
| **No** | 2 (6.9%) | 0 (0.0%) | | 4 (11.1%) | 3 (8.8%) | 4 (11.4%) | 6 (18.2%) | 10 (27.8%) | 14 (41.2%) |
| **P value** | 0.493 | | | 1.000 | | 0.507 | | 0.315 | |
|  | **SRS Score** | | | | | | | | |
|  | Less than sample median | | More than sample median | Less than sample median | More than sample median | Less than sample median | More than sample median | Less than sample median | More than sample median |
| **Yes** | 30 (96.8%) | | 28 (96.6%) | 38 (92.7%) | 33 (89.2%) | 33 (80.5%) | 33 (94.3%) | 31 (75.6%) | 22 (59.5%) |
| **No** | 1 (3.2%) | | 1 (3.4%) | 3 (7.3%) | 4 (10.8%) | 8 (19.5%) | 2 (5.7%) | 10 (24.4%) | 15 (40.5%) |
| **P value** | 1.000 | | | 0.702 | | 0.097 | | 0.150 | |
|  | **Health conditions at baseline** | | | | | | | | |
|  | Less than sample median | | More than sample median | Less than sample median | More than sample median | Less than sample median | More than sample median | Less than sample median | More than sample median |
| **Yes** | 27 (96.4%) | | 32 (97.0%) | 32 (88.9%) | 41 (93.2%) | 27 (77.1%) | 41 (95.3%) | 25 (69.4%) | 30 (68.2%) |
| **No** | 1 (3.6%) | | 1 (3.0%) | 4 (11.1%) | 3 (6.8%) | 8 (22.9%) | 2 (4.7%) | 11 (30.6%) | 14 (31.8%) |
| **P value** | 1.000 | | | 0.695 | | 0.037 | | 1.000 | |

Table S7 Carers and supporters’ views of the Health Check PAQ

|  | **Carers/supporters**, *N = 11* |
| --- | --- |
| **The Pre-appointment questionnaire** |  |
| Did you help the autistic person complete their Pre-appointment questionnaire? |  |
| Yes, completed on behalf of the autistic person | 4 (36%) |
| Yes, supported the autistic person to complete | 3 (27%) |
| No | 4 (36%) |
| If yes, what type of Pre-appointment questionnaire did you complete/help complete? |  |
| Paper version | 6 (86%) |
| Online version | 1 (14%) |
| Was the Pre-appointment questionnaire okay for you to complete or help complete? |  |
| Yes | 6 (86%) |
| No | 1 (14%) |
| Did thinking about the topics in the Pre-appointment questionnaire cause the autistic person any difficulties? |  |
| Yes | 4 (57%) |
| No | 2 (29%) |
| Missing | 1 (14%) |
| Did you think the Pre-appointment questionnaire was useful? |  |
| Yes | 7 (100%) |
| No | 0 (0%) |
| If yes, what was useful about it? |  |
| Sharing information about the autistic person’s communication before the appointment | 7 (100%) |
| Sharing information about the adjustments needed for the appointment | 7 (100%) |
| Sharing information about the autistic person’s current health concerns before the appointment | 5 (71%) |
| Sharing information about the autistic person’s general health and wellbeing before the appointment | 5 (71%) |
| Being able to write things down rather than having to talk to others | 4 (57%) |
| It covered all the things that were important to the autistic person | 4 (57%) |
| The autistic person didn’t have to answer as many questions at the appointment | 5 (71%) |
| Did the Pre-appointment questionnaire give enough opportunities to add in detail about the autistic person? |  |
| Yes | 7 (100%) |
| No | 0 (0%) |
| Time taken to complete the Pre-appointment questionnaire? |  |
| 31-45 minutes | 4 (57%) |
| 46-60 minutes | 2 (29%) |
| > 1 hour | 1 (14%) |
| How did you find the time taken to complete this? |  |
| About right | 5 (71%) |
| A little too long | 1 (14%) |
| Missing | 1 (14%) |

Table S8 Carers and supporters’ views of the Health Check appointment

|  | **Carers/supporters,**  *N = 11* |
| --- | --- |
| **The health check appointment** |  |
| Did you help to arrange the person’s health check appointment? |  |
| Yes | 6 (55%) |
| No | 5 (45%) |
| Do you think it was helpful to attend the health check appointment with the autistic person? |  |
| Yes | 11 (100%) |
| No |  |
| Did the autistic person request any adjustments for the health check appointment? |  |
| Yes | 6 (55%) |
| No | 3 (27%) |
| I don’t know | 2 (18%) |
| Were the requested adjustments made at the health check appointment? |  |
| Most or all of the adjustments I requested were made | 5 (83%) |
| None of the adjustments were made | 1 (17%) |
| Do you think the adjustments were helpful? |  |
| No |  |
| Yes | 5 (100%) |
| If yes, how were they helpful? |  |
| Helped the autistic person attend the appointment | 3 (60%) |
| Helped the autistic person give information to the GP/nurse | 4 (80%) |
| Helped the autistic person with waiting for their appointment | 2 (40%) |
| Helped the autistic person to remain calm and comfortable at their appointment | 5 (100%) |
| Helped the autistic person make good decisions about their health and healthcare | 1 (20%) |
| Helped make examinations, procedures or treatments more successful | 3 (60%) |
| Helped the autistic person to better understand or follow any advice | 2 (40%) |
| Other | 3 (60%) |
| Do you think the adjustments made the appointment better for the autistic person than their usual appointments at the GP practice? |  |
| Yes | 4 (80%) |
| No | 1 (20%) |
| Do you think the autistic person found it helpful for you to attend the health check appointment with them? |  |
| Yes | 11 (100%) |
| No | 0 (0%) |
| Would you attend another health check appointment with the autistic person in the future? |  |
| Yes | 11 (100%) |
| No | 0 (0%) |
| How often do you think the health check should be offered to autistic adults? |  |
| Every year | 11 (100%) |
| Every 2-3 years | 0 (0%) |
| Every 4-5 years | 0 (0%) |
| Do you think you learned anything from being involved in the health check appointment? |  |
| Yes | 9 (82%) |
| No | 1 (9%) |
| Missing | 1 (9%) |
| Since you attended the health check appointment with the autistic person, do you think there have been any improvements to your health or quality of life? |  |
| Yes | 2 (18%) |
| No | 9 (82%) |

Table S9 Clinicians’ views of the PAQ

|  | **Clinicians,**  *N =71* |
| --- | --- |
| **The health check summary report** |  |
| Pre-appointment questionnaire summary produced automatically |  |
| Yes | 28 (39%) |
| No | 35 (49%) |
| PAQ not returned by autistic adult | 6 (9%) |
| Unknown (completed by someone else) | 2 (3%) |
| Time spent preparing pre-appointment questionnaire summary |  |
| Numbers with response | 31 (89%) |
| Mean (SD) | 23.1 (16.7) |
| Median (IQR) | 15.0 (10.0, 30.0) |
| Range | 5.0, 60.0 |
| Time taken to read summary |  |
| Numbers with response | 64 (90%) |
| Mean (SD) | 10.1 (3.8) |
| Median (IQR) | 10.0 (10.0, 12.5) |
| Range | 0.0, 20.0 |
| Did you have enough time to read and understand the summary report before the appointment? |  |
| Numbers with response | 64 (90%) |
| Yes | 55 (86%) |
| No | 9 (14%) |
| Did the patient request adjustments in their summary report? |  |
| Yes | 31 (44%) |
| Yes, but this was raised at the start of HC appt | 3 (4%) |
| No | 33 (47%) |
| Participant did not return the PAQ | 4 (6%) |
| Were you able to offer the adjustments requested? |  |
| Yes, most or all | 28 (82%) |
| Yes, some of them | 5 (15%) |
| No | 1 (3%) |
| Did staff contact the patient to agree the adjustments to be implemented at the appointment? |  |
| Yes | 19 (56%) |
| No | 12 (35%) |
| The patient requested not to be contacted | 2 (6%) |
| Not sure | 1 (3%) |
| Time taken to contact patient and agree adjustments |  |
| Numbers with responses | 18 (95%) |
| Mean (SD) | 7.2 (4.3) |
| Median (IQR) | 5.0 (5.0, 10.0) |
| Range | 0.0, 20.0 |
| Time taken to implement requested adjustments |  |
| Numbers with responses | 29 (88%) |
| Mean (SD) | 4.0 (4.61) |
| Median (IQR) | 2.0 (0.0, 10.0) |
| Range | 0.0, 15.0 |
| Were any additional staff resources required to implement the requested adjustments? |  |
| Yes | 1 (3%) |
| No | 33 (97%) |
| Additional staff time required to implement adjustments (Mean (SD); Median (Range)) |  |
| Numbers with responses | 1 (1%) |
| Receptionist/administrator | 15.0 (.) ;  15.0 (15.0, 15.0) |

Table S10 Clinicians’ views of the health check appointment.

|  | **Clinicians,**  *N =71* |
| --- | --- |
| **The health check appointment** |  |
| Time taken to deliver the health check (minutes) |  |
| Numbers with responses | 70 (99%) |
| Mean (SD) | 58.4 (22.1) |
| Median (IQR) | 60.0 (45.0, 60.0) |
| Range | 30.0, 120.0 |
| If you did provide adjustments, did these make this appointment more useful than previous healthcare appointments with the patient? |  |
| Yes | 17 (52%) |
| No | 5 (15%) |
| Missing | 11 (33%) |
| Did you complete all sections of the health check during the appointment? |  |
| Yes, all sections | 14 (20%) |
| Completed most sections – included all that were relevant | 51 (72%) |
| Completed most sections – but missed some that were possibly relevant | 2 (3%) |
| No | 1 (1%) |
| Missing | 3 (4%) |
| Did you create a Health Action Plan? |  |
| Yes | 60 (85%) |
| No | 10 (14%) |
| Missing | 1 (1%) |
| If yes, did you give a copy of the Health Action Plan to the autistic person? |  |
| Yes | 46 (77%) |
| No | 12 (20%) |
| Missing | 2 (3%) |
| Time taken to update the patient notes after the health check took place (minutes) |  |
| Numbers with data | 69 (97%) |
| Mean (SD) | 13.57 (6.10) |
| Median (IQR) | 15.00 (10.00, 20.00) |
| Range | 5.00, 30.00 |

Table S11 Subgroup analyses according to practice size/environment of clinician reported evaluations

|  | **Were you able to offer the adjustments requested?** | | | | **Were any additional staff resources required to implement the requested adjustments?** | | | | **Did you create a health action plan?** | | | |
| --- | --- | --- | --- | --- | --- | --- | --- | --- | --- | --- | --- | --- |
|  | **Practice Size** | | | | | | | | | | | |
|  | Small | | Large | | Small | | Large | | Small | | Large | |
| **Yes** | 8 (100.0%) | | 22 (95.7%) | | 0 (0.0%) | | 1 (3.8%) | | 14 (63.6%) | | 45 (95.7%) | |
| **No** | 0 (0.0%) | | 1 (4.3%) | | 8 (100.0%) | | 25 (96.2%) | | 8 (36.4%) | | 2 (4.3%) | |
| **P value** | 1.000 | | | | 1.000 | | | | 0.001 | | | |
|  | **Practice environment** | | | | | | | | | | | |
|  | Urban | Rural | | Mixed | Urban | Rural | | Mixed | Urban | Rural | | Mixed |
| **Yes** | 20 (95.2%) | 4 (100.0%) | | 6 (100.0%) | 1 (4.8%) | 0 (0.0%) | | 0 (0.0%) | 40 (90.9%) | 6 (54.5%) | | 13 (92.9%) |
| **No** | 1 (4.8%) | 0 (0.0%) | | 0 (0.0%) | 20 (95.2%) | 7 (100.0%) | | 6 (100.0%) | 4 (9.1%) | 5 (45.5%) | | 1 (7.1%) |
| **P value** | 1.000 | | | | 1.000 | | | | 0.012 | | | |
