# Supplementary material for: Acceptability and Implementation of a Primary Care Health Check for Autistic People: Findings From Evaluation Questionnaires and Interviews
Source: Autism. 2026 Jun 16;30(8):1955–70. doi: 10.1177/13623613261433106 (PMC13392152; doi:10.1177/13623613261433106)
Supplement: sj-pdf-4-aut-10.1177_13623613261433106 – Supplemental material for Acceptability and Implementation of a Primary Care Health Check for Autistic People: Findings From Evaluation Questionnaires and Interviews [file sj-pdf-4-aut-10.1177_13623613261433106.pdf]

Health Checks for Autistic Adults Trial Procedure

Practice Randomised to Health Check Group

Screen potential participants and mailout study information pack

Consent and baseline assessment by research team or practice staff

Deliver Health Check

Post intervention outcome measures:
Health Check Evaluation Questionnaire
Interview (if selected)

Follow up outcome measures – 3, 6 and 9 months

9 months data collection from Health Records
